# Efficacy of Xianling Gubao capsule vs. its combination therapy in the treatment of primary osteoporosis: A network meta-analysis of randomized controlled trials

**DOI:** 10.1016/j.heliyon.2024.e29711

**Published:** 2024-04-21

**Authors:** Fushuang Yang, Tianyi Su, Zhenkun Liu, Fang Xia, Cheng Yu, Li Ma, Xin Su

**Affiliations:** a Children's Diagnosis and Treatment Center, The Affiliated Hospital to Changchun University of Chinese Medicine, China; b The Second Norman Bethune Hospital of Jilin University, China; c Department of Gynaecology, The First Clinical Hospital of Jilin Province Academy of Traditional Chinese Medicine, China; d College of Health Management, Changchun University of Chinese Medicine, China; e College of Basic Medical Sciences, Changchun University of Chinese Medicine, China

**Keywords:** Xianling Gubao capsule, Traditional Chinese medicine, Complementary and alternative medicine, Primary osteoporosis, Efficacy, Network meta-analysis

## Abstract

**Objective:**

This study aimed to evaluate the efficacy of the Xianling Gubao (XLGB) capsule alone and its combination therapy in primary osteoporosis (POP).

**Methods:**

Databases including PubMed, Embase, Cochrane Library, Web of Science, CNKI, Wanfang Data, VIP, and SinoMed were searched from their inception to January 16, 2024, for randomized controlled trials (RCTs) investigating the XLGB treatment for POP. A network meta-analysis (NMA) was performed to evaluate the efficacy and safety of multiple interventions in the treatment of POP. The Cochrane risk-of-bias tool was used to assess the quality of RCTs included in the meta-analysis. Software Stata (version 15.0) was used for statistical analysis. The surface under the cumulative ranking curve (SUCRA) method was used to present the findings from this NMA numerically and graphically by ranking multiple interventions.

**Results:**

A total of 107 RCTs were included in the meta-analysis, involving 10,032 participants and 21 interventions. Meta-analysis showed that XLGB + calcium (Ca) + calcitonin (99.9 %) was the most desirable treatment option for improving clinical efficacy. XLGB + Ca + bisphosphonate (BP) was most effective for improving bone mineral density (BMD) at the lumbar spine, femoral neck BMD, and serum bone Gla protein (BGP). SUCRA values for improving these three outcome measures by XLGB + Ca + BP were 87.4 %, 77.2 %, and 84.3 %, respectively. XLGB + calcitonin was the optimal option in terms of safety evaluation and improving visual analogue scale (VAS), with the SUCRA values being 89.6 % and 94.9 %, respectively.

**Conclusions:**

The XLGB combination therapy is a desirable option for treating POP as it can effectively improve the therapeutic effects, BMD, and serum BGP, as well as relieve pain in patients with POP.

## 1 Introduction

People with osteoporosis have less bone mass and experience microarchitectural deterioration of bone tissue, making them susceptible to bone fractures [1]. It has been estimated that over 200 million people are bothered by osteoporosis, which is responsible for 8.9 million cases of fracture [2]. In China, the prevalence of this disease is around 7 % among adults, 22.5 % among males aged 50 or above, and 50.1 % among females [3]. It is estimated that the number of people experiencing hip fractures in China will rise from 41,000 in 2015 to more than 1 million in 2050 [4]. Osteoporosis has been identified by the World Health Organization (WHO) as a significant global public health burden given the high morbidity, disability, mortality, and poor quality of life associated with this disease [5]. Particularly, the incidence of osteoporosis increases with age, and therefore the potential number of people developing osteoporosis and the associated social burden may increase in the future as the population ages [6].

Primary osteoporosis (POP) is caused by natural changes to bone density rather than other underlying conditions. It can be divided into postmenopausal osteoporosis (type I) and senile osteoporosis (type II). Reducing bone loss and correcting bone remodeling imbalance are fundamental to the treatment of POP. In addition to lifestyle adjustments (reducing alcohol intake, smoking cessation, healthy diet, enhanced nutrition, and physical exercise), the current basic therapies for the prevention and treatment of POP include the use of vitamin D (VD) and calcium (Ca) supplements [7]. However, medical interventions are needed for patients with POP at higher risk of fracture and can be split into two categories: 1) Antiresorptive agents, including bisphosphonates (BPs), oestrogen, calcitonin, and selective oestrogen receptor modulators (SERM). They inhibit bone resorption for maintaining bone density; 2) Anabolic agents, including teriparatide and abaloparatide, which stimulate bone formation to increase bone density [8]. Despite the good efficacy of these agents in clinical practice, adverse reactions associated with these agents arouse great concern [9,10]. In China, some patients seek help from traditional Chinese medicine (TCM) in addition to the abovementioned treatments.

According to the TCM theory, most bone diseases are associated with pain caused by a deficiency of the kidney and blood stasis in the meridians. Xianling Gubao (XLGB) capsule, the use of which was approved by the China Food and Drug Administration (now National Medical Products Administration, NMPA) in 2002, is the cornerstone of Chinese proprietary medicines for treating osteoporosis. XLGB, composed of *Epimedium alpinum* L, *Radix dipsaci*, *Psoralea corylifolia* L, *Rehmannia glutinosa* (Gaertn.) DC, *Salvia miltiorrhiza* Bunge, and *Anemarrhena asphodeloides* Bunge, can support liver and kidney functions, promote blood circulation, and remove the meridian obstruction, thus strengthening the muscles and bones [11,12]. Studies have found that the XLGB capsule can regulate the differentiation and proliferation of osteoclasts and osteoblasts through various signaling pathways to treat POP [13,14]. Although the XLGB capsule is widely applied in China for the treatment of POP, its efficacy compared with other medications or XLGB combination therapy remains controversial given a lack of evidence-based studies. Luo [15] conducted a network meta-analysis (NMA) to evaluate the clinical efficacy of XLGB and XLGB combined with two or more drugs for treating postmenopausal osteoporosis. Despite the higher prevalence of fracture among postmenopausal women with POP, fracture in older men is also an important public health concern [16]. Therefore, a meta-analysis was conducted in the present study to compare the clinical efficacy of XLGB with other treatment options, providing a reference for the appropriate application of XLGB in clinical practice.

## 2 Methods

This meta-analysis was reported following the Preferred Reporting Items for Systematic Reviews and Meta-Analyses 2020 (the PRISMA 2020) guideline [17]. The present study has been registered on PROSPERO (CRD42022357443).

### 2.1 Literature search

Databases including China National Knowledge Infrastructure (CNKI), Wanfang Data, VIP, SinoMed (Chinese biomedical literature service system), PubMed, Embase, Cochrane Library, and Web of Science were searched from their inception till January 16, 2024, for randomized controlled trials (RCTs) investigating the treatment for POP with XLGB. Medical Subject Headings (MeSH) and keywords were used for the literature search. MeSH is “Osteoporosis” [Mesh]. Keywords are listed as follows: “Osteoporosis” OR “Osteoporoses” OR “pathologic decalcification” in combination with “xianling gubao” OR “Xianlinggubao” OR “XLGB” OR “xian-ling-gu-bao”. Corresponding translation terms (in Chinese) were used for literature search in Chinese databases including CNKI, Wanfang Data, VIP, and SinoMed. In addition, we searched the references in studies of interest to look for potentially relevant studies. The literature search strategy is presented in Supplementary Material 1.

### 2.2 Inclusion criteria

1) Participants: Patients diagnosed with POP, including postmenopausal osteoporosis (type I, which usually refers to osteoporosis that occurs within 5–10 years after menopause) and senile osteoporosis (type II, which generally refers to osteoporosis occurring after the age of 70) [18]. 2) Interventions: Participants in the experimental group were treated with XLGB alone or XLGB combined with other drugs, while those in the control group received no treatment, placebo, or conventional Western medicine. 3) Outcome measures: Primary outcome measures were clinical efficacy, bone mineral density (BMD) at the lumbar spine, BMD at the femoral neck, serum bone Gla protein (BGP), visual analogue scale (VAS) results, and adverse reactions. At least one abovementioned outcome measure was investigated in each of the included studies. 4) Study type: RCTs were included. 5) Each type of intervention should be investigated by at least two included studies.

### 2.3 Exclusion criteria

Studies were excluded if they 1) were reviews, conference abstracts, letters, guidelines, case reports, pathological mechanisms, etc., 2) investigated participants with POP and other skeletal disorders, 3) reported incomplete or incorrect data, 4) overlap each other in terms of targeted participants, and 5) were duplicate publications.

### 2.4 Literature screening and data extraction

Two researchers independently screened studies obtained from the databases, extracted data from the included studies, and cross-checked the data. In case of disagreement, the corresponding author was consulted, and a consensus was finally reached. We tried our best to get in contact with the authors of corresponding studies to obtain the missing information if any. Studies retrieved were first imported into the software EndNote X9 (version 19.0) (Clarivate Analytics, Philadelphia, USA) to remove duplicate publications, followed by subsequent removal of irrelevant articles. Then, eligible studies were determined after a full-text review. Information extracted from eligible studies included: 1) basic characteristics of eligible studies: title, first author, year of publication, country of publication, etc.; 2) baseline characteristics of participants: sample size, age, sex, etc.; 3) interventions: details, course of treatment, etc.; 4) key parameters for risk-of-bias assessment; 5) outcome measures and measurement of the outcomes.

### 2.5 Risk of bias assessment

Cochrane's risk-of-bias assessment tool was used to assess the quality across the included studies [19] based on bias arising from the randomization process, bias due to deviations from intended interventions, bias due to missing outcome data, bias in the measurement of the outcome, and bias due to the selective reporting.

### 2.6 Statistical analysis

Package mvmeta and network in software Stata 15.0 (StataCorp, College Station, TX, United States) were employed for NMA [20,21]. Dichotomous variables were expressed as the relative ratio (RR) with a corresponding 95 % confidence interval (CI) and measurement data were presented as mean difference (MD) with 95 % CI. The relationship between interventions was presented using network plots, with each node representing an intervention. The size of a node was directly proportional to the sample size of each intervention. The edge between the two nodes indicated a direct comparison between the two interventions, and the thickness of each line was positively correlated with the number of randomized comparisons between the two corresponding interventions.

If closed loops were formed in a network plot, inconsistency in the NMA was assessed. If no closed loop was formed, the consistency model was used for data analysis. Overall assessment of inconsistency was followed by the node-splitting method to assess local inconsistency (based on the difference coefficients between direct and indirect comparison) [22]. P value < 0.05 indicated significant inconsistency, and the source of inconsistency was explored. The surface under the cumulative ranking (SUCRA) curve method was used to rank interventions in terms of efficacy. SUCRA values ranged from 0 % to 100 %. The closer the value was to 100 %, the more likely that an intervention was to be among the most desirable treatments. Comparison-adjusted funnel plots were drawn to assess the risk of publication bias across included RCTs.

## 3 Results

### 3.1 Literature search

**Fig. 1:**
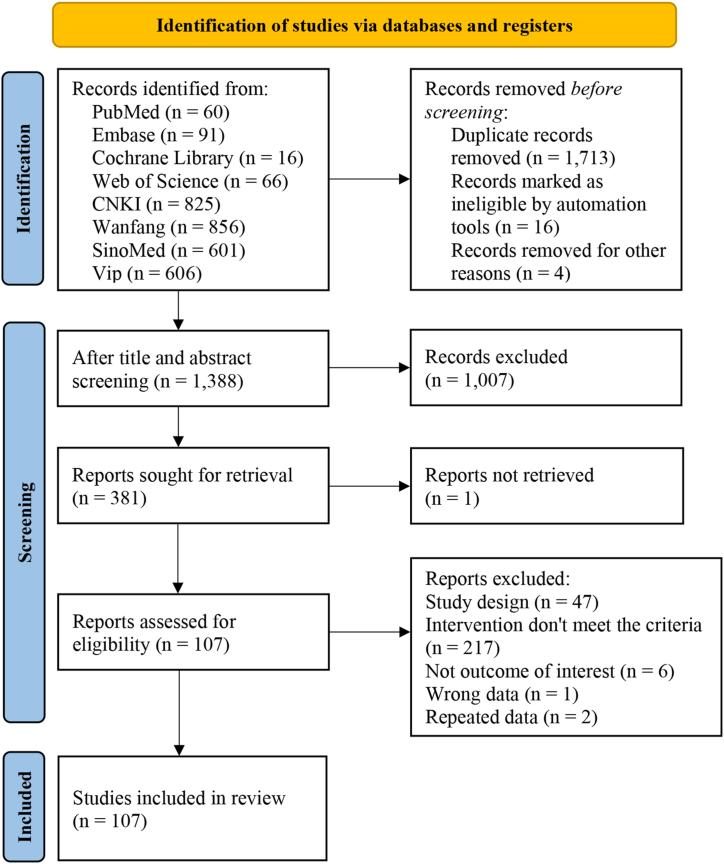
Flow diagram of the study selection process.

A total of 3,121 articles were obtained from a literature search in databases, including 825 articles from CNKI, 856 from Wanfang, 606 from VIP, 601 from SinoMed, 60 from PubMed, 91 from Embase, 16 from Cochrane Library, and 66 from Web of Science. Duplicate publications were removed first, followed by a screening of titles and abstracts. Then, the full texts of 381 articles were reviewed based on predefined inclusion and exclusion criteria. Finally, 107 studies [[23], [24], [25], [26], [27], [28], [29], [30], [31], [32], [33], [34], [35], [36], [37], [38], [39], [40], [41], [42], [43], [44], [45], [46], [47], [48], [49], [50], [51], [52], [53], [54], [55], [56], [57], [58], [59], [60], [61], [62], [63], [64], [65], [66], [67], [68], [69], [70], [71], [72], [73], [74], [75], [76], [77], [78], [79], [80], [81], [82], [83], [84], [85], [86], [87], [88], [89], [90], [91], [92], [93], [94], [95], [96], [97], [98], [99], [100], [101], [102], [103], [104], [105], [106], [107], [108], [109], [110], [111], [112], [113], [114], [115], [116], [117], [118], [119], [120], [121], [122], [123], [124], [125], [126], [127], [128], [129]] were included in the NMA. The process of screening studies is presented in Fig. 1.

### 3.2 Study characteristics

Eligible studies were published between 2004 and 2023. Among them, 2 studies [51,105] were published in English, and the rest were in Chinese. A total of 10,032 participants were investigated in these studies, with 5,041 in the treatment group and 4,991 in the control group. There were 2,100 cases of postmenopausal osteoporosis (type I) and 6,225 cases of senile osteoporosis (type II). The remaining 1,707 participants were diagnosed with POP while the type of POP was not clarified. The included studies all reported comparability between groups in terms of age, sex, and course of the disease. Two or more drugs in combination were investigated by 88 studies, and XLGB alone was evaluated by 19 studies. The course of treatment and the duration of follow-up were 1–36 months. Twenty-one interventions were investigated in these studies as Ca, XLGB, BP, VD, Calcitonin, XLGB + Ca, XLGB + BP, XLGB + BP + calcitonin, BP + calcitonin, XLGB + calcitonin, XLGB + Ca + BP, Ca + BP, Ca + VD + BP, XLGB + Ca + VD + BP, Ca + VD + calcitonin, XLGB + Ca + VD + calcitonin, Ca + calcitonin, XLGB + Ca + VD, Ca + VD, XLGB + VD, and XLGB + Ca + calcitonin. Characteristics of the included studies are presented in Table 1.

**Table 1:** The characteristics of the included studies.

No.	Included studies	Treatment group 1				Treatment group 2				Treatment course (months)
		Treatment	Sample size (male/female)	Age (years)	Course of disease (years)	Treatment	Sample size (male/female)	Age (years)	Course of disease (years)	
1	Zhao [126,127]	BP	43 (26/17)	69.73 ± 3.18	4.12 ± 1.63	XLGB + BP	43 (25/18)	70.61 ± 3.26	4.51 ± 1.47	6
2	Zhao [126,127]	BP	38 (38/0)	81.8 ± 5.7	3.3 ± 0.6	XLGB + BP	38 (38/0)	81.9 ± 5.6	3.2 ± 0.7	6
3	Wu et al. [130]	Ca + BP	50 (23/27)	61.64 ± 10.32	5.24 ± 1.94	XLGB	50 (22/28)	62.16 ± 11.25	5.97 ± 2.03	6
4	Dong [129]	Ca	39 (0/39)	50.3 ± 2.5	5.08 ± 1.18	XLGB	39 (0/39)	50.15 ± 2.45	5.05 ± 1.2	1
5	Gao [23]	Ca + BP	34 (18/16)	70.25 ± 6.84	6.35 ± 2.01	XLGB + Ca + BP	34 (17/17)	70.16 ± 6.48	6.51 ± 2.14	6
6	Pan et al. [24]	Ca + BP	45 (22/23)	67.65 ± 6.18	4.86 ± 1.02	XLGB + Ca + BP	45 (21/24)	68.47 ± 5.95	5.02 ± 1.19	6
7	Wang et al. [25]	Ca + BP	80 (47/33)	61.5 ± 7.4	3.76 ± 2.51	XLGB + Ca + BP	80 (49/31)	62.9 ± 8.2	4.21 ± 1.49	3
8	Fu et al. [69]	Ca + BP	41 (22/19)	69.45 ± 2.01	5.17 ± 1.01	XLGB + Ca + BP	41 (23/18)	69.40 ± 2.23	5.24 ± 0.98	12
9	Liu et al. [27]	Ca	34 (0/34)	73 ± 12.16	1.26 ± 0.17	XLGB	34 (0/34)	73.5 ± 12.25	1.22 ± 0.23	1
10	Zhang et al. [131], [28]	Ca + BP	28 (0/28)	67.64 ± 3.56	5.68 ± 1.45	XLGB + Ca + BP	28 (0/28)	68.34 ± 3.25	5.53 ± 1.35	12
11	Feng [29]	BP	42 (23/19)	/	/	XLGB	42 (22/20)	/	/	3
12	Li [30]	BP	75 (41/34)	67.88 ± 7.93	4.1 ± 0.44	XLGB + BP	75 (45/30)	68.07 ± 8.57	4.16 ± 0.52	4
13	Ma et al. [31]	Ca + BP	69 (0/69)	68.33 ± 3.62	6.47 ± 1.45	XLGB + Ca + BP	69 (0/69)	68.51 ± 3.73	6.36 ± 1.54	3
14	Xie [32]	Calcitonin	25 (25/0)	76.42 ± 3.54	5.12 ± 2.13	XLGB + calcitonin	25 (13/12)	76.28 ± 3.35	5.09 ± 2.61	3
15	Cai et al. [33]	BP	63 (0/63)	59.66 ± 7.34	/	XLGB + BP	63 (0/63)	58.79 ± 8.82	/	6
16	Zhou [34]	Ca + BP	63 (0/63)	66.72 ± 8.24	6.34 ± 1.41	XLGB + Ca + BP	63 (0/63)	66.79 ± 8.16	6.28 ± 1.36	6
17	Li [35]	Ca	51 (0/51)	61.8 ± 2.5	/	XLGB + Ca	51 (0/51)	62.2 ± 2.3	/	12
18	Liu et al. [36]	Ca + BP	30 (19/11)	61.92	4.18	XLGB + Ca + BP	30 (18/12)	61.89	4.21	6
19	Kang et al. [37]	BP + calcitonin	32 (11/21)	66.91 ± 2.16	6.85 ± 1.14	XLGB + BP + calcitonin	32 (9/23)	66.87 ± 2.13	6.87 ± 1.12	3
20	Wang et al. [38]	BP	46 (25/21)	68.75 ± 3.56	5.32 ± 2.16	XLGB + BP	46 (23/23)	67.95 ± 3.74	5.64 ± 2.25	3
21	Guo et al. [39]	BP + calcitonin	42 (18/24)	70.39 ± 5.07	6.31 ± 1.72	XLGB + BP + calcitonin	42 (17/25)	70.43 ± 5.11	6.33 ± 1.7	3
22	Zhang et al. [40]	BP	43 (16/27)	71.54 ± 4.32	7.54 ± 1.29	XLGB + BP	43 (15/28)	71.93 ± 3.71	7.90 ± 1.54	12
23	Zhao et al. [41]	BP	56 (32/24)	70.1 ± 2.6	/	XLGB + BP	56 (29/27)	71.2 ± 2.4	/	2
24	Chen et al. [113]	BP	41 (23/18)	73.2 ± 8.4	1.2 ± 0.36	XLGB	46 (27/29)	73.8 ± 8.1	1.5 ± 0.42	1
25	Dang [43]	Ca	39 (14/25)	57.87 ± 10.94	6.63 ± 2.08	XLGB + Ca	39 (12/27)	60.13 ± 11.98	6.78 ± 2.19	3
26	Guo [44]	Ca + calcitonin	155 (70/85)	58.6 ± 3.5	6.89 ± 1.16	XLGB + Ca + calcitonin	156 (72/84)	58.5 ± 3.4	6.56 ± 1.28	3
27	Li et al. [45]	Ca + BP	62 (32/30)	75 ± 4	5.3 ± 2	XLGB + Ca + BP	62 (31/31)	76 ± 3	5 ± 2.3	6
28	Zhu [46]	BP	31 (0/31)	63.65 ± 8.22	/	XLGB + calcitonin	31 (0/31)	62.55 ± 6.63	/	3
29	Ye et al. [47]	BP	67 (18/49)	59.37 ± 6.25	6.03 ± 1.39	XLGB + BP	67 (19/48)	58.93 ± 6.51	6.17 ± 1.54	6
30	Yan [48]	Ca + BP	34 (14/20)	63.5 ± 4.9	/	XLGB + Ca + BP	34 (15/19)	63.2 ± 5.2	/	6
31	Li et al. [49]	Ca + BP	30 (13/17)	58.73 ± 4.21	6.39 ± 2.75	XLGB + Ca + BP	30 (12/18)	59.56 ± 4.87	6.74 ± 3.04	6
32	Zeng et al. [50]	BP + calcitonin	51 (23/28)	65.02 ± 10.64	6.77 ± 1.62	XLGB + BP + calcitonin	51 (24/27)	64.91 ± 10.35	6.72 ± 1.54	3
33	Guo et al. [51]	Ca + VD	30 (21/9)	67.95 ± 3.05	4.6 ± 1.9	XLGB + Ca + VD	30 (17/13)	69.16 ± 3.58	4.7 ± 2.3	3
34	Qu et al. [52]	Ca	20 (0/20)	84 ± 5	/	XLGB + Ca	20 (0/20)	84 ± 5	/	6
35	Li [53]	Ca	43 (0/43)	61.25 ± 2.47	5.04 ± 1.18	XLGB + Ca	43 (0/43)	61.12 ± 2.42	5.02 ± 1.13	1
36	Liang [54]	Ca	40	60.61 ± 6.14	/	XLGB + Ca	40	60.61 ± 6.14	/	6
37	Liu [55]	BP	33 (18/15)	67.3 ± 3.3	/	XLGB + BP	33 (20/13)	68.6 ± 3.2	/	1
38	Hou et al. [56]	BP	29	/	/	XLGB + BP	29	/	/	6
39	Zhang [57]	Ca	50	61.85 ± 6.42	/	XLGB + Ca	50	61.85 ± 6.42	/	1
40	Cao et al. [58]	Ca + BP	25 (12/13)	57 ± 0.7	1.5	XLGB + Ca + BP	25 (12/13)	56 ± 0.9	1.5	12
41	Song [59]	Calcitonin	31 (0/31)	55.21 ± 2.97	/	XLGB + calcitonin	31 (0/31)	56 ± 3.16	/	6
42	Luo [60]	BP	30 (18/12)	60 ± 3	5.4 ± 1.2	XLGB	30 (19/11)	62 ± 3	5.0 ± 1.1	6
43	Wang et al. [61]	BP + calcitonin	60 (24/36)	55.6 ± 10.4	5.6 ± 2.8	XLGB + BP + calcitonin	60 (27/33)	55.5 ± 9.8	4.9 ± 2.1	3
44	Lin et al. [62]	Ca	30 (19/11)	61.47 ± 5.92	/	XLGB	30 (20/10)	61.25 ± 6.14	/	1
45	Yuan et al. [63]	BP	34 (4/30)	62.61 ± 3.43	/	XLGB + BP	34 (3/31)	62.32 ± 3.15	/	2
46	Zhang [64]	BP + calcitonin	50 (10/40)	70.15 ± 6.30	4.88 ± 1.02	XLGB + BP + calcitonin	50 (20/30)	70.23 ± 6.20	4.85 ± 1.02	12
47	Hou et al. [65]	Ca + VD + BP	56 (20/36)	69.13 ± 10.23	6.51 ± 1.39	XLGB + Ca + VD + BP	56 (23/33)	67.73 ± 11.03	6.19 ± 1.50	3
48	Liu et al. [66]	Ca + BP	65	60.21 ± 5.29	4.06 ± 1.55	XLGB + Ca + BP	67	60.21 ± 5.29	4.06 ± 1.55	6
49	Li [67]	VD	30 (14/16)	70.67 ± 5.30	/	XLGB + VD	28 (11/17)	69.25 ± 8.53	/	6
50	Deng [68]	Calcitonin	56 (22/34)	63.9 ± 1.5	/	XLGB + calcitonin	56 (23/33)	64.5 ± 1.3	/	1
51	Fu et al. [69]	Ca + BP	150 (64/86)	73.95 ± 15.54	4.75 ± 2.31	XLGB + Ca + BP	150 (61/89)	74.31 ± 16.28	4.81 ± 2.09	12
52	Jin [70]	Ca + BP	60	58.8 ± 5.1	/	XLGB + Ca + BP	60	58.4 ± 4.2	/	6
53	Zhang [71]	BP	101 (49/52)	61.3 ± 3.6	/	XLGB + BP	102 (52/50)	61.4 ± 3.7	/	3
54	Zheng [72]	Ca	49 (9/40)	55.33 ± 11.49	/	XLGB	49 (10/39)	56.61 ± 10.36	/	4
55	Zhou et al. [73]	Ca + BP	71 (30/41)	58.2 ± 1.1	5.7 ± 0.9	XLGB + Ca + BP	71 (27/44)	58.6 ± 1.3	5.8 ± 0.8	6
56	Chen et al. [74]	BP	25 (0/25)	62.5 ± 6.8	/	XLGB + calcitonin	30 (0/30)	63.3 ± 6.2	/	3
57	Han [75]	BP	55 (34/21)	71.9 ± 8.1	6 ± 1.8	XLGB + Ca + BP	55 (35/20)	72.1 ± 8.4	6.1 ± 1.9	6
58	Li [76]	Ca	34 (0/34)	58.5 ± 0.3	/	XLGB + BP	34 (0/34)	58.2 ± 0.3	/	6
59	Chen et al. [78]	Ca + VD	30 (0/30)	54.86 ± 5.19	/	XLGB + Ca + VD	30 (0/30)	56.45 ± 5.33	/	6
60	Chen et al. [78]	BP + calcitonin	59 (23/36)	72.1 ± 11.2	5.25 ± 1.93	XLGB + BP + calcitonin	59 (21/38)	71.6 ± 10.3	5.98 ± 2.04	3
61	Qin et al. [79]	Ca + BP	80	71.5 ± 6.3	6.4 ± 2.1	XLGB	80	71.5 ± 6.3	6.4 ± 2.1	1
62	Bao et al. [80]	Ca + BP	32	75.19 ± 5.07	4.62 ± 1.35	XLGB + Ca + BP	32	75.19 ± 5.07	4.62 ± 1.35	12
63	He et al. [81]	BP	39 (22/17)	68.85 ± 4.45	/	XLGB + BP	40 (17/23)	67.50 ± 4.16	/	36
64	Zeng et al. [82]	BP + calcitonin	39 (15/24)	73.98 ± 5.87	4.69 ± 1.18	XLGB + BP + calcitonin	40 (18/22)	75.01 ± 6.14	4.82 ± 1.09	12
65	Bao et al. [83]	Ca + BP	70 (29/41)	58.1 ± 1	5.9 ± 1.1	XLGB + Ca + BP	70 (26/44)	59 ± 1	5.7 ± 1	6
66	Lu [84]	Ca + VD + calcitonin	60 (20/40)	68.6	/	XLGB + Ca + VD + calcitonin	60 (22/38)	68.5	/	2
67	Wang [85]	Ca	42	60.85 ± 6.32	/	XLGB	42	60.85 ± 6.32	/	1
68	Wei [86]	Calcitonin	72	67.54 ± 8.53	/	XLGB + calcitonin	72	67.54 ± 8.53	/	2
69	Xu et al. [87]	BP	45	61.81 ± 2.69	3.06 ± 0.51	XLGB + BP	45	61.81 ± 2.69	3.06 ± 0.51	6
70	Zhang et al. [88]	BP	45 (17/28)	67.91 ± 8.74	6.93 ± 3.02	XLGB + BP	45 (19/26)	68.35 ± 9.56	7.47 ± 2.51	6
71	Jin et al. [89]	Ca	80 (31/49)	69.84 ± 9.02	5.97 ± 3.14	XLGB	80 (34/46)	70.25 ± 8.39	6.24 ± 2.94	3
72	Lin [90]	Ca + calcitonin	60 (38/22)	71.84 ± 2.99	/	XLGB + Ca + calcitonin	60 (40/20)	72.32 ± 2.97	/	2
73	Liu [91]	BP	31 (16/15)	/	/	XLGB + BP	31 (17/14)	/	/	6
74	Peng et al. [92]	Calcitonin	42 (14/28)	68.8 ± 6.4	/	XLGB + calcitonin	42 (15/27)	68.6 ± 6.5	/	/
75	Qiao et al. [93]	Ca	60 (0/60)	61.27 ± 2.17	5.13 ± 2.01	XLGB	60 (0/60)	61.27 ± 2.17	5.13 ± 2.01	1
76	Ouyang et al. [94]	Ca	28	/	/	XLGB + Ca	30	/	/	3
77	Wu [95]	Ca	42	60.85 ± 6.32	/	XLGB	42	60.85 ± 6.32	/	1
78	Shi et al. [96]	Ca	46 (18/28)	60.38 ± 9.05	7.02 ± 5.15	XLGB + Ca	46 (16/30)	63.34 ± 8.54	7.54 ± 5.35	3
79	Peng et al. [97]	Calcitonin	15	55∼82	/	XLGB	15	55∼82	/	1
80	Zhuang et al. [98]	BP	32 (0/32)	60.34 ± 2.35	/	XLGB + BP	32 (0/32)	59.78 ± 2.17	/	6
81	Wei [99]	BP	48 (6/42)	72	3.8	XLGB + BP	50 (3/47)	72	3.8	6
82	Wu et al. [100]	Ca	29	77.9	/	XLGB + Ca	29	77.9	/	3
83	Yu et al. [101]	Calcitonin	34	82.8 ± 4.8	/	XLGB + calcitonin	35	81.4 ± 4.7	/	6
84	Wang et al. [102]	Calcitonin	30	55∼75	/	XLGB + calcitonin	30	55∼75	/	1
85	Luo [103]	Ca + BP	42 (22/20)	52∼71	/	XLGB + Ca + BP	42 (23/19)	50∼72	/	12
86	Gong et al. [104]	BP	30 (4/26)	52∼90	/	XLGB + BP	30 (5/25)	52∼90	/	6
87	Zhu et al. [105]	Ca + VD	61 (0/61)	64.9 ± 6.0	/	XLGB + Ca + VD	61 (0/61)	65.4 ± 6.3	/	12
88	Jin [106]	Ca + calcitonin	36 (21/15)	/	/	XLGB + Ca + calcitonin	36 (19/17)	/	/	3
89	Li et al. [107]	Ca + calcitonin	32 (17/15)	60.5	/	XLGB + Ca + calcitonin	32 (14/18)	61.4	/	6
90	Zhang et al. [108]	Ca + calcitonin	44 (17/27)	70.6 ± 5.62	/	XLGB + Ca + calcitonin	46 (16/30)	69.2 ± 5.49	/	2
91	Yuan et al. [109]	Ca	40 (19/21)	58.3 ± 6.9	5.4 ± 3.3	XLGB + Ca	40 (23/17)	59.4 ± 7.35	5.6 ± 3.2	2
92	Liu et al. [110]	Ca	45 (0/45)	59.8 ± 8.6	6.5 ± 1.9	XLGB + Ca	45 (0/45)	60.3 ± 10.2	6.1 ± 2.3	6
93	Li [111]	Ca + calcitonin	30 (14/16)	/	/	XLGB + Ca + calcitonin	30 (12/18)	/	/	1
94	Wu [112]	Ca	33 (0/33)	56.3 ± 3.5	/	XLGB	37 (0/37)	55.1 ± 2.9	/	1
95	Dong [113]	Calcitonin	53 (0/53)	60.71 ± 9.07	/	XLGB + calcitonin	54 (0/54)	61.43 ± 8.22	/	3
96	Liang [114]	Ca	43 (29/14)	63.9 ± 4.8	/	XLGB + Ca	43 (31/12)	64.3 ± 5.1	/	6
97	Liu [115]	Ca	48	≥60	/	XLGB + Ca	48	≥60	/	6
98	Zhang [116]	Ca	28 (6/22)	67.3	/	XLGB + Ca	28 (9/19)	68.4	/	3
99	Liang [117]	Ca	60	/	/	XLGB	60	/	/	3
100	Xu et al. [118]	BP	52 (0/52)	58.2 ± 2.8	/	XLGB + BP	52 (0/52)	58.2 ± 2.8	/	6
101	Song et al. [119]	BP	50 (18/32)	61.7	/	XLGB	50 (15/35)	63.65	/	3
102	Wu et al. [120]	Ca	23 (0/23)	55.6 ± 3.9	/	XLGB + Ca + BP	33 (0/33)	54.2 ± 4.2	/	1
103	Yang et al. [121]	Ca	41 (10/31)	60.4	8	XLGB + Ca + calcitonin	43 (14/29)	62.5	7	3
104	Shang et al. [122]	Ca	30 (0/30)	58.3 ± 3.4	/	XLGB + Ca	30 (0/30)	58.3 ± 3.4	/	6
105	Ren et al. [123]	Calcitonin	60 (26/34)	59.2 ± 6.3	/	XLGB + calcitonin	60 (22/38)	57.4 ± 5.6	/	1
106	Wu et al. [124]	Ca	34 (0/34)	56.4 ± 4.6	/	XLGB	34 (0/34)	55.6 ± 4.3	/	12
107	Zhang et al. [125]	Ca	50 (0/50)	57.2 ± 3.1	/	XLGB	62 (0/62)	57.2 ± 3.1	/	12

### 3.3 Quality of the included studies

**Fig. 2:**
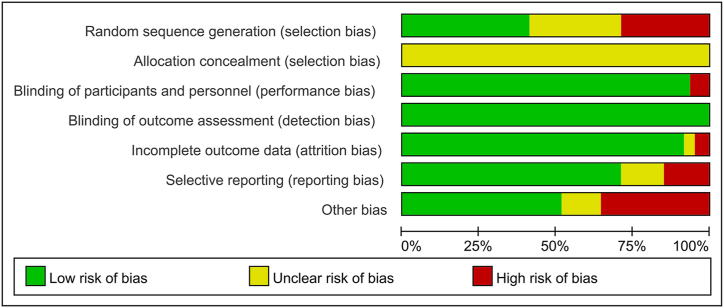
Risk of bias.

All the included studies were rated as having an unclear or high risk of bias, mainly attributed to a lack of randomization sequence concealment and absence of blinding. Right random allocation methods were described in 44 studies such as random number tables, random numbers generated by a computer, and drawing lots. High-risk randomization methods such as randomization by patient admission data, admission number, and interventions were reported in 31 studies. The remaining 32 studies did not offer clear information on random allocation. No studies described the concealment of the scheme or blinding methods. In the case of blinding of participants, subjective results such as VAS scores may be biased, but objective results such as BMD and BGP are unlikely to be biased. All studies had a low risk of bias regarding incomplete results and selective reporting. The results of the risk-of-bias assessment across the included studies are shown in Fig. 2.

## 4 Network meta-analysis

### 4.1 Network plot

**Fig. 3:**
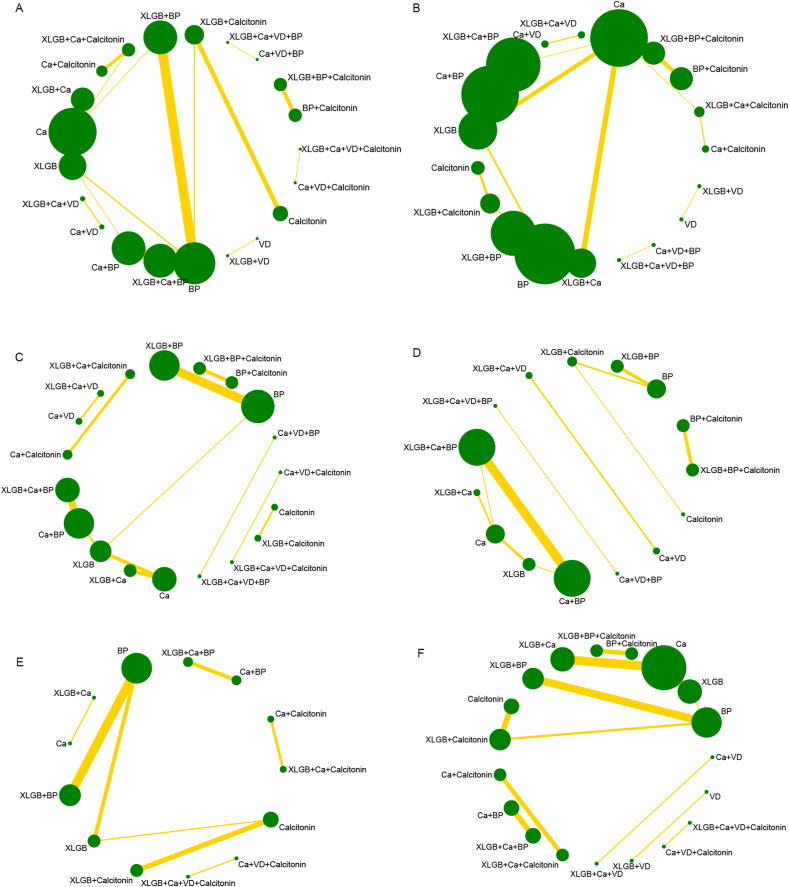
Network diagrams depicting the direct evidence used in the network meta-analysis. Network diagram of clinical efficacy (A), lumbar BMD (B), femoral neck BMD (C), BGP (D), VAS (E), and adverse reactions (F).

Among 21 interventions that were investigated in the included studies, 11 evaluated XLGB. Fig. 3 presents the network plots for six outcome measures, with each node representing an intervention and the node size being directly proportional to the sample size associated with the intervention that the node stands for. Edges between nodes mean direct comparisons between pairs of interventions, with line thickness in direct proportion to the number of trials comparing the two corresponding interventions.

### 4.2 Inconsistency tests

In the network plot for clinical efficacy, one closed loop was formed by 21 interventions. Overall assessment of inconsistency showed good agreement between the direct and indirect sources of evidence (*P* = 0.757), and assessment of local inconsistency revealed no inconsistency (*P* > 0.05). As for BMD at the lumbar spine, a closed loop was formed by 19 interventions. Overall assessment of inconsistency showed good agreement between the direct and indirect sources of evidence (*P* = 0.418), and assessment of local inconsistency revealed no inconsistency (*P* > 0.05). One closed loop was formed by 15 interventions for BGP, with good agreement between the direct and indirect comparisons of multiple interventions (*P* = 0.873), and an assessment of local inconsistency revealed no inconsistency (*P* > 0.05).

### 4.3 Network meta-analysis of multiple outcome measures

Comparisons of 55 pairs of interventions contributed to the evaluation of efficacy, with 33 pairs of comparison indicating statistical significance, among which 5 pairs involved the comparison of basic treatment versus XLGB combination therapy, including calcitonin versus XLGB + calcitonin (RR = 0.82, 95 % CI [0.76, 0.88]), BP versus XLGB + BP (RR = 0.76, 95 % CI [0.60, 0.98]), Ca + calcitonin versus XLGB + Ca + calcitonin (RR = 0.94, 95 % CI [0.90, 0.98]), Ca + BP versus XLGB + Ca + BP (RR = 0.84, 95 % CI [0.81, 0.88]), and Ca versus XLGB + Ca (RR = 0.78, 95 % CI [0.73, 0.83]). XLGB alone was superior to three interventions in the comparisons, namely calcitonin versus XLGB (RR = 0.77, 95 % CI [0.66, 0.90]), BP versus XLGB (RR = 0.85, 95 % CI [0.73, 0.99]), and Ca + calcitonin versus XLGB (RR = 0.82, 95 % CI [0.77, 0.87]), and the difference was statistically significant as shown in Table 2.

**Table 2:** Bottom left side: Network meta-analysis of clinical efficacy. Upper right: Network meta-analysis of adverse reactions.

Calcitonin	4.16 (1.26,13.73)	–	–	4.85 (1.06,22.31)	0.94 (0.57,1.55)	2.53 (0.65,9.87)	–	–	3.94 (0.81,19.17)	1.96 (0.48,7.91)
0.99 (0.83,1.17)	BP	–	–	1.06 (0.42,2.65)	**0.23 (0.08,0.67)**	0.61 (0.32,1.17)	–	–	0.95 (0.34,2.68)	**0.47 (0.23,0.97)**
										
**0.44 (0.29,0.67)**	**0.45 (0.30,0.66)**	Ca + calcitonin	–	–	–	–	–	–	–	–
0.89 (0.67,1.18)	0.90 (0.73,1.13)	**2.02 (1.37,2.98)**	Ca + BP	–	–	–	–	–	–	–
0.92 (0.72,1.17)	0.93 (0.79,1.10)	**2.09 (1.47,2.97)**	1.03 (0.88,1.22)	Ca	**0.21 (0.05,0.88)**	0.57 (0.19,1.77)	–	–	0.89 (0.55,1.45)	**0.44 (0.25,0.78)**
**0.82 (0.76,0.88)**	**0.83 (0.71,0.97)**	**1.86 (1.22,2.82)**	0.92 (0.70,1.20)	0.89 (0.70,1.11)	XLGB + calcitonin	2.69 (0.76,9.54)	–	–	4.18 (0.93,18.79)	2.08 (0.56,7.66)
0.75 (0.56,1.02)	**0.76 (0.60,0.98)**	**1.71 (1.15,2.54)**	0.85 (0.66,1.08)	**0.82 (0.68,0.98)**	0.92 (0.69,1.24)	XLGB + BP	–	–	1.55 (0.46,5.29)	0.77 (0.29,2.04)
**0.41 (0.27,0.63)**	**0.42 (0.29,0.62)**	**0.94 (0.90,0.98)**	**0.47 (0.32,0.68)**	**0.45 (0.32,0.64)**	**0.51 (0.33,0.77)**	**0.55 (0.37,0.81)**	XLGB + Ca + calcitonin	–	–	–
0.75 (0.57,1.00)	**0.76 (0.61,0.95)**	**1.71 (1.16,2.52)**	**0.84 (0.81,0.88)**	**0.82 (0.69,0.97)**	0.92 (0.70,1.21)	1.00 (0.78,1.28)	**1.81 (1.23,2.68)**	XLGB + Ca + BP	–	–
**0.72 (0.56,0.92)**	**0.73 (0.61,0.87)**	**1.64 (1.15,2.34)**	**0.81 (0.68,0.97)**	**0.78 (0.73,0.83)**	0.88 (0.70,1.12)	0.96 (0.79,1.16)	**1.74 (1.22,2.48)**	0.96 (0.80,1.15)	XLGB + Ca	0.50 (0.24,1.05)
**0.77 (0.66,0.90)**	**0.85 (0.73,0.99)**	**0.82 (0.77,0.87)**	0.93 (0.74,1.16)	1.01 (0.83,1.22)	0.92 (0.72,1.18)	**1.83 (1.28,2.61)**	**1.61 (1.26,2.38)**	0.93 (0.70,1.22)	1.05 (0.96,1.15)	XLGB

Comparisons of 36 pairs of interventions offered data on BMD at the lumbar spine, with 9 pairs of comparison indicating statistical significance, among which 3 pairs involved the comparison of basic treatment versus XLGB combination therapy, including BP versus XLGB + BP (MD = −0.81, 95 % CI [−1.07, −0.54]), Ca + BP versus XLGB + Ca + BP (MD = −1.35, 95 % CI [−2.69, −0.01]), and Ca versus XLGB + Ca (MD = −0.47, 95 % CI [−0.81, −0.13]). XLGB alone was superior to two interventions in the comparisons, namely BP versus XLGB (MD = −0.85, 95 % CI [−1.23, −0.46]), and Ca versus XLGB (MD = −0.61, 95 % CI [−0.94, −0.28]), and the difference was statistically significant, as shown in Table 3.

**Table 3:** Bottom left side: Network meta-analysis of lumbar BMD. Upper right: Network meta-analysis of femoral neck BMD.

BP	−0.40 (−1.66,0.86)	−0.09 (−1.24,1.07)	–	0.45 (0.12,0.78)	–	0.55 (−0.76,1.87)	0.36 (−0.92,1.63)	0.30 (−0.73,1.34)
−0.21 (−1.11,0.69)	Ca + BP	0.32 (−0.57,1.21)	–	0.85 (−0.45,2.15)	–	**0.96 (0.58,1.34)**	0.76 (−0.28,1.80)	0.71 (0.02,1.43)
0.13 (−0.57,0.83)	0.34 (−0.44,1.12)	Ca	–	0.53 (−0.67,1.73)	–	0.64 (−0.33,1.61)	**0.75 (0.22,1.28)**	0.39 (−0.13,0.91)
−0.46 (−1.20,0.27)	−0.25 (−1.41,0.91)	−0.59 (−1.61,0.42)	XLGB + calcitonin	–	–	–	–	–
**−0.81 (-1.07,-0.54)**	−0.60 (−1.54,0.34)	**−0.94 (-1.69,-0.19)**	−0.35 (−1.13,0.43)	XLGB + BP	–	0.11 (−1.25,1.46)	−0.09 (−1.41,1.22)	−0.14 (−1.23,0.94)
−0.31 (−1.53,0.91)	−0.10 (−1.37,1.17)	−0.44 (−1.43,0.56)	0.15 (−1.27,1.58)	0.50 (−0.75,1.75)	XLGB + Ca + calcitonin	–	–	–
**−1.56 (-2.86,-0.26)**	**−1.35 (-2.69,-0.01)**	**−1.69 (-2.78,-0.60)**	−1.10 (−2.59,0.39)	−0.75 (−2.08,0.57)	−1.25 (−2.73,0.23)	XLGB + Ca + BP	−0.20 (−1.30,0.91)	−0.25 (−1.07,0.57)
−0.34 (−1.12,0.44)	−0.13 (−0.98,0.72)	**−0.47 (-0.81,-0.13)**	0.12 (−0.95,1.19)	0.47 (−0.35,1.29)	−0.03 (−1.08,1.02)	**1.22 (0.07,2.36)**	XLGB + Ca	−0.05 (−0.80,0.69)
**−0.85 (-1.23,-0.46)**	−0.51 (−1.19,0.17)	**−0.61 (-0.94,-0.28)**	0.09 (−0.56,0.73)	−0.41 (−1.48,0.66)	0.84 (−0.32,2.00)	−0.57 (−1.93,0.80)	−0.38 (−0.89,0.13)	XLGB

Comparisons of 21 pairs of interventions provided data for analysis of BMD at the femoral neck. XLGB + Ca + BP was significantly more effective than Ca + BP regarding the improvement in BMD at the femoral neck (MD = 0.96, 95 % CI [0.58, 1.34]), BP + XLGB was superior to BP alone (MD = 0.45, 95 % CI [0.12, 0.78]), and Ca + XLGB was superior to Ca alone (MD = 0.75, 95 % CI [0.22, 1.28]), with a statistically significant difference. No statistically significant difference was observed in the comparison of the remaining pairs of interventions, as shown in Table 3.

Comparisons of 10 pairs of interventions contributed to the analysis of BGP that were associated with multiple interventions. XLGB + Ca was more effective in improving BGP compared with Ca alone (MD = 1.84, 95 % CI [0.63, 3.05]), XLGB + Ca + BP was superior to Ca + BP (MD = 2.07, 95 % CI [0.92, 3.20]), and XLGB alone was better than Ca + BP regarding improvement in BGP (MD = 1.62, 95 % CI [0.66, 2.59]), with a significant difference. No statistically significant difference was observed in the comparison of the remaining pairs of interventions, as shown in Table 4.

**Table 4:** Bottom left side: Network meta-analysis of BGP. Upper right: Network meta-analysis of VAS.

Calcitonin	1.07 (−0.58,2.73)	−1.32 (−1.99,-0.65)	−0.24 (−1.97,1.50)	−0.12 (−1.58,1.34)
Ca + BP	BP	**−2.39 (-4.17,-0.61)**	**−1.31 (-1.83,-0.79)**	**−1.19 (-1.98,-0.41)**
0.65 (−1.23,2.53)	Ca	XLGB + calcitonin	1.08 (−0.78,2.94)	1.20 (−0.41,2.80)
**−2.07 (-3.20,-0.92)**	−2.72 (−8.56,3.12)	XLGB + Ca + BP	XLGB + BP	0.12 (−0.82,1.06)
−1.19 (−3.42,1.05)	**−1.84 (-3.05, -0.63)**	0.88 (−5.09,6.84)	XLGB + Ca	XLGB
**−1.62 (-2.59, -0.66)**	1.10 (−4.82,7.01)	−0.50 (−6.66,5.65)	0.22 (−1.33,1.77)	XLGB

Comparisons of 10 pairs of interventions in terms of improving VAS showed that XLGB + calcitonin effectively reduced VAS scores compared with calcitonin alone (MD = −1.32, 95 % CI [−1.99, −0.65]), XLGB + BP was better than BP alone (MD = −1.31, 95 % CI [−1.83, −0.79]), and XLGB was superior to BP (MD = −1.19, 95 % CI [−1.98, −0.41]) in reducing VAS scores, with a significant difference. No statistically significant difference was found between comparisons of the remaining pairs of interventions, as shown in Table 4.

As for adverse reactions, comparisons of 21 pairs of interventions were analyzed, and 3 pairs involved the comparison of XLGB combination therapy versus basic treatment, including XLGB + calcitonin versus calcitonin (RR = 0.94, 95 % CI [0.57, 1.55]), XLGB + BP versus BP (RR = 0.61, 95 % CI [0.32, 1.17]), and XLGB + Ca versus Ca (RR = 0.89, 95 % CI [0.55, 1.45]), and there was no statistically significant difference, indicating that XLGB combination therapy did not increase the risk of adverse reactions. In addition, XLGB alone showed statistical significance compared with BP (RR = 0.47, 95 % CI [0.23, 0.97]) or Ca (RR = 0.44, 95 % CI [0.25, 0.78]), indicating that XLGB was safer, as shown in Table 2.

### 4.4 Probability ranking based on SUCRA values

**Fig. 4:**
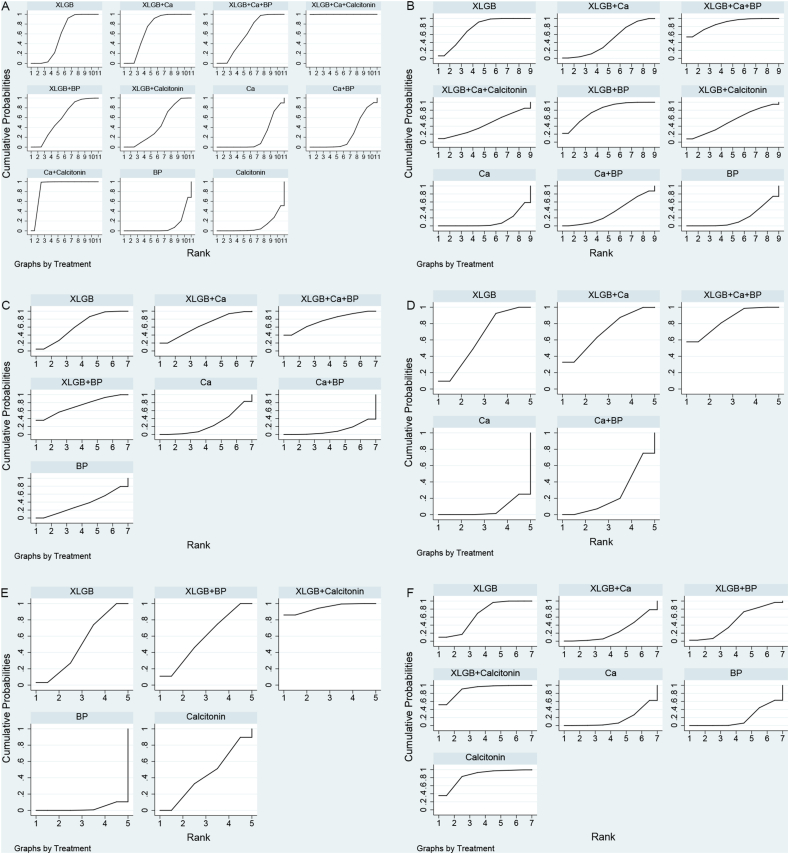
SUCRA value and probability ranking chart. SUCRA value and probability ranking of clinical efficacy (A), lumbar BMD (B), femoral neck BMD (C), BGP (D), VAS (E), and adverse reactions (F).

Table 5 presents the probability ranking of multiple interventions in improving six outcome measures. In terms of improving clinical efficacy, XLGB + Ca + calcitonin (99.9 %), Ca + calcitonin (89.9 %), and XLGB + Ca (70.5 %) ranked among the most desirable treatment options. XLGB + Ca + BP (87.4 %), XLGB + BP (78.5 %), and XLGB (74.7 %) were the most effective approaches to improving BMD in the lumbar spine. The three best interventions for improving BMD at the femoral neck were XLGB + Ca + BP (77.2 %), XLGB + BP (71.2 %), and XLGB + Ca (65.8 %). The three best options for improving serum BGP levels were XLGB + Ca + BP (84.3 %), XLGB + Ca (70.8 %), and XLGB (62.7 %). In terms of improving VAS results, the three best interventions for pain relief were XLGB + calcitonin (94.9 %), XLGB + BP (57.9 %), and XLGB (51.0 %). As for safety, XLGB + calcitonin (89.6 %), calcitonin (84.4 %), and XLGB (65.4 %) were among the safest treatment options. The SUCRA value and probability ranking are shown in Fig. 4.

**Table 5:** Rank of the efficacy of included treatments.

Outcome indicator	Treatment methods											
	Clinical effect		BMD of lumbar		BMD of femoral neck		BGP		VAS		Adverse reactions	
	SUCRA value	Rank	SUCRA value	Rank	SUCRA value	Rank	SUCRA value	Rank	SUCRA value	Rank	SUCRA value	Rank
XLGB	57.8	6	74.7	3	62.1	4	62.7	3	51.0	3	65.4	3
Ca	20.5	9	11.3	9	27.5	6	6.6	5	–	–	16.1	7
BP	9.6	11	18.8	8	34.2	5	–	–	2.8	5	18.9	6
Calcitonin	9.9	10	–	–	–	–	–	–	43.4	4	84.4	2
XLGB + Ca	70.5	3	44.2	6	65.8	3	70.8	2	–	–	25.9	5
XLGB + BP	59.2	5	78.5	2	71.2	2	–	–	57.9	2	49.6	4
XLGB + calcitonin	45.6	7	53.1	4	–	–	–	–	94.9	1	89.6	1
XLGB + Ca + BP	60.6	4	87.4	1	77.2	1	84.3	1	–	–	–	–
Ca + BP	26.5	8	35.1	7	12.1	7	25.6	4	–	–	–	–
Ca + calcitonin	89.9	2	–	–	–	–	–	–	–	–	–	–
XLGB + Ca + calcitonin	99.9	1	45.8	5	–	–	–	–	–	–	–	–

### 4.5 Cluster analysis

**Fig. 5:**
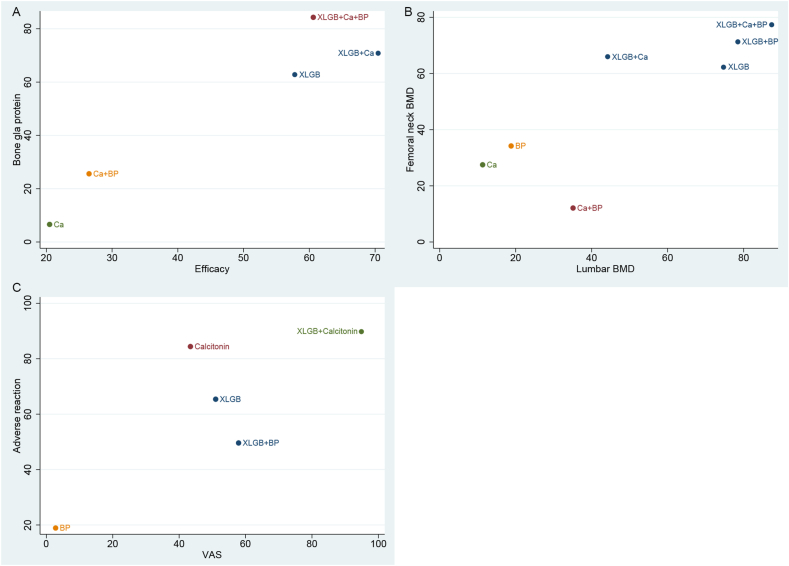
Cluster analysis.

Cluster analysis was carried out based on SUCRA values and indicated that XLGB + Ca + BP was the most effective treatment for improving efficacy and serum BGP, while XLGB + Ca + BP, XLGB + BP, and XLGB alone were most effective in improving BMD at the lumbar spine or femoral neck. XLGB + calcitonin was superior to other regimens with respect to pain relief and safety, as shown in Fig. 5.

### 4.6 Publication bias

**Fig. 6:**
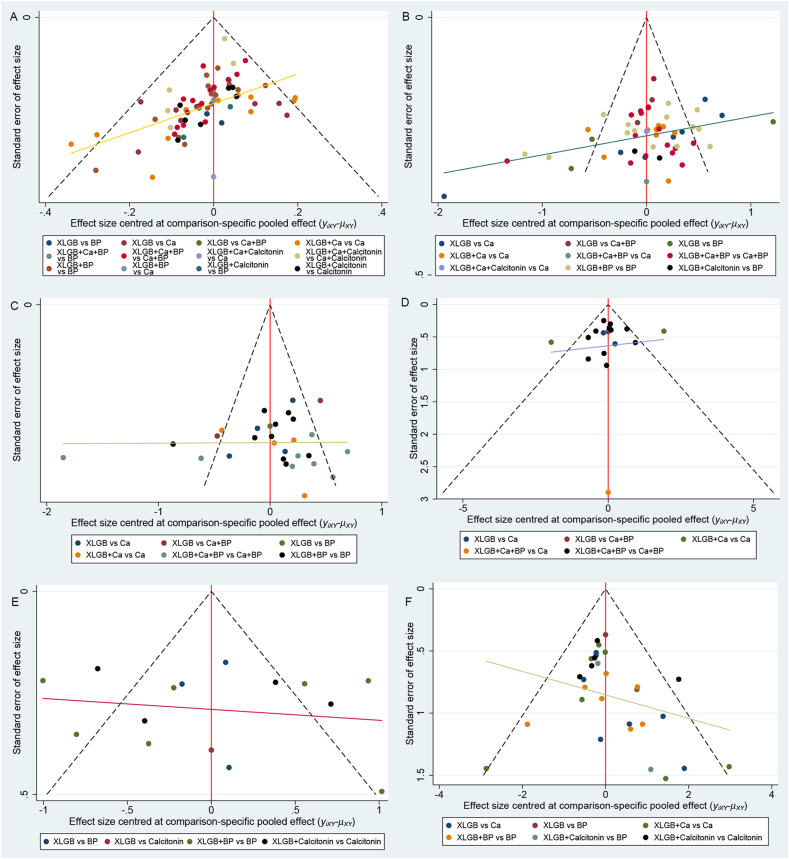
Comparison-adjusted funnel plots. Comparison-adjusted funnel plots of clinical efficacy (A), lumbar BMD (B), femoral neck BMD (C), BGP (D), VAS (E), and adverse reactions (F).

The comparison-adjusted funnel plots of multiple outcome measures showed that there was a low risk of publication bias among the included studies with respect to clinical efficacy, BMD at the lumbar spine, BGP, VAS results, and adverse reactions, as shown in Fig. 6.

## 5 Discussion

XLGB is an over-the-counter (OTC) traditional Chinese medicine preparation approved by NMPA for the treatment of osteoporosis and fractures [132]. XLGB was recommended to treat osteoporosis by the Guidelines for Clinical Application of Chinese Patent Medicine in the Treatment of Osteoporosis (2021) [133], and the Guidelines for the Diagnosis and Treatment of Primary Osteoporosis (2022) [18], and its effectiveness in treating POP has been confirmed by many clinical trials [27,29,42,60,62]. Original studies and conventional meta-analyses only included direct comparisons of XLGB with other treatment options. However, indirect evidence is also needed for a comprehensive understanding of the efficacy of XLGB. Therefore, an NMA was conducted in the present study to pool evidence for XLGB or XLGB combination therapy to indirectly compare the efficacy of multiple options for the treatment of POP. A total of 107 RCTs were included in the NMA, involving 10,032 participants and 21 treatments. Analysis results showed that XLGB + Ca + calcitonin was most effective in improving clinical efficacy for treating POP, XLGB + Ca + BP was the most desirable treatment option regarding improving BMD at the lumbar spine or femoral neck and increasing serum BGP, and XLGB + calcitonin was the best in terms of pain relief and safety. Overall, the combination of XLGB with other treatments outperformed the basic regimen regarding improving clinical efficacy, BMD, and BGP and alleviating pain.

XLGB consists of six types of Chinese herbs: *Epimedium alpinum* L, *Radix dipsaci*, *Psoralea corylifolia* L, *Rehmannia glutinosa* (Gaertn.) DC, *Salvia miltiorrhiza* Bunge, and *Anemarrhena asphodeloides* Bunge, with *Epimedium* being the essential ingredient. A meta-analysis [134] revealed that *Epimedium* has satisfactory therapeutic effects on treating osteoporosis as it can regulate bone metabolism, improve BMD, and relieve pain. *Icariin*, the main component of *Epimedium*, can not only promote osteogenic differentiation *in vivo* but also inhibit osteoclast generation and bone resorption activity [135]. In a postmenopausal rodent model induced by ovariectomy, *Radix dipsaci* prevented bone loss and deterioration of trabecular microstructure and increased bone strength, and this was possibly associated with changes in bone remodeling and decreased levels of bone turnover markers [136]. *Psoralea corylifolia* L and *Rehmannia glutinosa* (Gaertn.) DC are important Chinese herbs for the treatment of osteoporosis, both of which can promote osteogenic activity and improve bone absorption by osteoclasts to alleviate osteoporosis [137,138]. *Salvia miltiorrhiza* Bunge, which targets different pathways in the bone remodeling cycle, including activation of osteoblasts, inhibition of osteoclast generation, and degradation of collagen by cathepsin K, has been proven in clinical practice to have good anti-osteoporosis potential [139]. Lee et al. [140] found that extracts from *Salvia miltiorrhiza* Bunge improved trabecular bone loss in naturally postmenopausal and ovariectomized rats by inhibiting bone resorption and osteoclast differentiation and were conducive to correcting abnormal levels of osteocalcin, bone alkaline phosphatase (BALP), and receptor activator of nuclear factor κ B (RANKL) that are strongly associated with bone resorption. Some researchers [141] investigated the effects of compound A. asphodeloides Bunge (AAB) in *Anemarrhena asphodeloides* Bunge on osteoporosis and the potential mechanism of AAB in bone remodeling. The results showed that AAB reversed the decrease of BMD and bone mineral content, significantly inhibited osteoclast formation, and improved osteoporosis, suggesting that *Anemarrhena asphodeloide*s Bunge may be a potential candidate for treating osteoporosis.

The present study showed that XLGB combination therapy was superior to the basic therapies concerning improvement in clinical efficacy, BMD, BGP, and alleviation of pain. Besides, XLGB alone outperformed some other treatment options. These findings are consistent with the conclusions of many previous studies. Zhang [142] found that drug-containing serum of XLGB capsule promoted the differentiation and proliferation of mouse embryonic osteoblast progenitor cells, and this was possibly attributed to the activation of mitogen activated protein kinases (MAPK) signaling pathway and up-regulation of expression of osteoblast-related factors, indicating that XLGB accelerates the proliferation of osteoblasts. The animal experiment conducted by Ren [131] revealed that XLGB increased BMD and serum BGP levels, relieved microstructural defect of fracture end, and promoted healing of broken bones, and this may be explained by up-regulated mRNA and protein expression of osteoprotectin and down-regulated mRNA and protein expression of RANKL ligand. Chen [143] pointed out that XLGB might inhibit the apoptosis of osteoblasts and promote their bone formation to treat osteoporosis. A clinical study by Wu [130] showed that XLGB had good efficacy in the treatment of osteoporosis as it could improve BMD and bone metabolism. Effective pain relief by XLGB combination therapy may be related to the analgesic effect of *Salvia miltiorrhiza* Bunge in XLGB, and this finding is supported by previous studies. Some studies [144,145] have demonstrated that tanshinones, the main chemical component of *Salvia miltiorrhiza* Bunge, has a good analgesic effect and can effectively relieve pain by raising the pain threshold.

The present study has some limitations. First, studies with small sample sizes that failed to clarify the criteria of sample size estimation dominated the included studies, possibly undermining the reliability of the related results. Second, none of the included studies described allocation concealment and blinding, affecting their methodological quality. Third, subtypes of POP (type Ⅰ or Ⅱ) were not reported for some participants in the included studies, and thus subgroup analysis was not performed based on the subtypes of POP. Fourth, publication bias was observed with respect to BMD at the femoral neck, and the course of treatment and the duration of follow-up differed between the included studies. These factors may bias the results of the meta-analysis. Additionally, as most of the participants were Asian people, the findings of the present study should be applied to the general population with caution.

## 6 Conclusion

XLGB combination therapy is a desirable option for treating POP as it can effectively improve clinical efficacy, BMD, and serum BGP, as well as relieve pain. More high-quality RCTs are needed in the future to validate this conclusion, given the quality of the included studies in this NMA.

## Funding

10.13039/501100013061Jilin Provincial Science and Technology Development Plan Project (No.202104048YY).

The fund was not involved in any study design, data collection, analysis and interpretation, report writing, or article submission for publication.

## Data availability statement

The data that support the findings of this study are available from the corresponding author upon reasonable request.

Has data associated with your study been deposited into a publicly available repository?

Data included in article/supp. material/referenced in article.

## Ethics approval and consent to participate

Not applicable.

## CRediT authorship contribution statement

**Fushuang Yang:** Conceptualization, Data curation, Formal analysis, Funding acquisition, Writing – original draft, Writing – review & editing. **Tianyi Su:** Investigation, Methodology, Project administration, Resources, Visualization, Writing – original draft, Writing – review & editing. **Zhenkun Liu:** Software, Validation, Writing – original draft, Writing – review & editing. **Fang Xia:** Conceptualization, Visualization, Writing – original draft, Writing – review & editing. **Cheng Yu:** Formal analysis, Writing – original draft, Writing – review & editing. **Li Ma:** Funding acquisition, Writing – original draft, Writing – review & editing. **Xin Su:** Project administration, Resources, Software, Supervision, Writing – original draft, Writing – review & editing.

## Declaration of competing interest

The authors declare that they have no known competing financial interests or personal relationships that could have appeared to influence the work reported in this paper.
