# Supplementary material for: Efficacy of Xianling Gubao capsule vs. its combination therapy in the treatment of primary osteoporosis: A network meta-analysis of randomized controlled trials
Source: Heliyon. 2024 Apr 21;10(9):e29711. doi: 10.1016/j.heliyon.2024.e29711 (PMC11066608; doi:10.1016/j.heliyon.2024.e29711)
Supplement: Multimedia component 1 [file mmc1.docx]

**Supplementary material 1 Search strategy**

**PubMed**

| **Search** | **Query** | **Results** |
| --- | --- | --- |
| 1 | "Osteoporosis"[Mesh] | 63,879 |
| 2 | ((Osteoporosis[Title/Abstract]) OR (Osteoporoses[Title/Abstract])) OR (pathologic decalcification[Title/Abstract]) | 86,233 |
| 3 | (((xianling gubao[Title/Abstract]) OR (Xianlinggubao[Title/Abstract])) OR (XLGB[Title/Abstract])) OR (xian-ling-gu-bao[Title/Abstract]) | 89 |
| 4 | ("Osteoporosis"[Mesh]) OR (((Osteoporosis[Title/Abstract]) OR (Osteoporoses[Title/Abstract])) OR (pathologic decalcification[Title/Abstract])) | 104,224 |
| 5 | ((((xianling gubao[Title/Abstract]) OR (Xianlinggubao[Title/Abstract])) OR (XLGB[Title/Abstract])) OR (xian-ling-gu-bao[Title/Abstract])) AND (("Osteoporosis"[Mesh]) OR (((Osteoporosis[Title/Abstract]) OR (Osteoporoses[Title/Abstract])) OR (pathologic decalcification[Title/Abstract]))) | 60 |

**Web of Science**

| **Search** | **Query** | **Results** |
| --- | --- | --- |
| 1 | TS=(Osteoporosis) OR TS=(Osteoporoses) OR TS=(pathologic decalcification) | 157,232 |
| 2 | TS=(xianling gubao) OR TS=(Xianlinggubao) OR TS=(XLGB) OR TS=(xian-ling-gu-bao) | 99 |
| 3 | #1 AND #2 | 66 |

**Embase**

| **Search** | **Query** | **Results** |
| --- | --- | --- |
| 1 | 'osteoporosis'/exp | 203,354 |
| 2 | 'xianlinggubao'/exp | 58 |
| 3 | 'xianling gubao':ab,ti OR xianlinggubao:ab,ti OR xlgb:ab,ti OR 'xian ling gu bao':ab,ti | 132 |
| 4 | osteoporosis:ab,ti OR osteoporoses:ab,ti OR 'pathologic decalcification':ab,ti | 126,546 |
| 5 | #1 OR #4 | 203,376 |
| 6 | #2 OR #3 | 139 |
| 7 | #5 AND #6 | 91 |

**Cochrane Library**

| **Search** | **Query** | **Results** |
| --- | --- | --- |
| 1 | MeSH descriptor: [Osteoporosis] explode all trees | 5,827 |
| 2 | (Osteoporosis):ti,ab,kw OR (Osteoporoses):ti,ab,kw OR (pathologic decalcification):ti,ab,kw | 12,187 |
| 3 | #1 or #2 | 12,187 |
| 4 | (xianling gubao):ti,ab,kw OR (Xianlinggubao):ti,ab,kw OR (XLGB):ti,ab,kw OR (xian-ling-gu-bao):ti,ab,kw | 26 |
| 5 | #3 and #4 | 16 |
